# Social Return on Investment Analysis of the Health Precinct Community Hub for Chronic Conditions

**DOI:** 10.3390/ijerph17145249

**Published:** 2020-07-21

**Authors:** Carys Jones, Ned Hartfiel, Paul Brocklehurst, Mary Lynch, Rhiannon Tudor Edwards

**Affiliations:** 1 Centre for Health Economics and Medicines Evaluation, Bangor University, Bangor LL57 2PZ, UK; ned.hartfiel@bangor.ac.uk (N.H.); m.lynch@bangor.ac.uk (M.L.); r.t.edwards@bangor.ac.uk (R.T.E.); 2 North Wales Organisation for Randomised Trials in Health, Bangor University, Bangor LL57 2PZ, UK; p.brocklehurst@bangor.ac.uk

**Keywords:** Social Return on Investment (SROI), social prescribing, physical activity

## Abstract

Local governments and Health Boards are seeking to develop integrated services to promote well-being. Social participation and physical activity are key in promoting well-being for older people. The Health Precinct is a community hub in North Wales that people with chronic conditions are referred to through social prescribing. To improve community-based assets there is a need to understand and evidence the social value they generate. Data collection took place October 2017–September 2019. Social Return on Investment (SROI) analysis was used to evaluate the Health Precinct. Stakeholders included participants aged 55+, participants’ families, staff, the National Health Service and local government. Participants’ health and well-being data were collected upon referral and four months later using the EQ-5D-5L, Campaign to End Loneliness Scale and the Rosenberg Self-Esteem Scale. Family members completed questionnaires at four months. Baseline data were collected for 159 participants. Follow-up data were available for 66 participants and 38 family members. The value of inputs was £55,389 (attendance fees, staffing, equipment, overheads), and the value of resulting benefits was £281,010; leading to a base case SROI ratio of £5.07 of social value generated for every £1 invested. Sensitivity analysis yielded estimates of between 2.60:1 and 5.16:1.

## 1. Introduction

### 1.1. Population Ageing and Chronic Conditions in Wales

Wales has the highest number of older people as a proportion of its population in the United Kingdom, with 27.1% of the population aged 60 and above [1]. This is projected to increase to 30.9% by 2040. Chronic conditions such as heart disease, cancer and respiratory disease are the leading causes of premature deaths in Wales [2]. The prevalence of chronic conditions increases with age, with two-thirds of over 65’s in Wales reporting having at least one chronic condition [2]. Modifiable risk factors for these conditions include physical inactivity, unhealthy diets, and alcohol and substance misuse [3]. Currently, National Health Service (NHS) expenditure in Wales is approximately £6.5 billion annually, or £2091 per person [4]. With the number of older people expected to rise, and therefore the number of people with chronic conditions rising, the impact will increase pressure on the NHS, with public expenditure on health and social care likely to struggle to meet demands.

### 1.2. Social Participation and Physical Activity for Older People

Life expectancy in the UK has increased steadily since the 19th century; however, healthy life expectancy has not risen at the same rate leading to the population living longer but spending more years in poorer health [5]. Socio-economic inequalities are a factor, with people living in more affluent areas in the South of the UK living for longer and in better health than people living in the North [5]. One of society’s remaining challenges is to address social determinants that cause poor health, e.g., loneliness, access to affordable and nutritious food, and safety in public spaces. These factors can cause poor health just as much as a physical condition such as a broken leg, but they require a social rather than a medical approach.

Local governments and Health Boards in Wales are being encouraged to work together to identify need and develop integrated services. Promoting well-being is a core component of the national outcomes framework in the new Social Services and Well-being (Wales) Act [6], and promoting social participation and physical activity are seen to be key in promoting the well-being of older people [7,8,9]. Social participation has been shown to have an inverse relationship with mortality [10] and is a factor in reducing the risk of depression, cognitive and motor decline and functional disability in older people [11,12]. Social prescribing is a practice where health professionals refer people to non-clinical services in the local area, often provided by the third sector (i.e. the voluntary, community and social enterprise sector) e.g., gardening schemes, sports groups or art groups. These services support people to take more control of their health and well-being through providing opportunities to learn new skills and form relationships with others in their community. It is a way of addressing health needs using a holistic approach which encompasses environmental, physical, functional and social aspects of quality of life [13].

### 1.3. The Health Precinct

Based in Colwyn Bay, North Wales, the Health Precinct is a partnership between Conwy County Borough Council, Betsi Cadwaladr University Health Board (BCUHB), and Public Health Wales. Facilities at the site include a public leisure centre, a hydrotherapy pool, tennis courts, a bowling green, a boating lake, an indoor football pitch, a running track and a rugby pitch. However, the approach of the Health Precinct goes beyond the physical amenities on site. People with chronic conditions are referred to it through social prescribing. A treatment plan will be developed after a multi-disciplinary assessment is undertaken. The plan is typically 16 weeks long and includes achievable exercise goals, along with Physiotherapy, Occupational Therapy, or Nursing advice. The theory is that removing barriers to exercise, such as offering services in a community setting rather than hospital or clinic, will promote co-production and self-management of conditions.

The target population for the Health Precinct is people of all ages, but to date it has been predominantly used by older people. The most frequent reasons for referral are issues with mobility and balance, musculoskeletal conditions e.g., arthritis and joint stiffness, heart conditions, weight loss, COPD and asthma. People with chronic conditions, living in the appropriate catchment area, are referred to the Health Precinct by a health professional. Social care teams are also able to sign-post people for referral. The most common pathways that people are referred to are Lifestyle Management, National Exercise Referral Scheme (NERS) and Cardiac Rehabilitation.

As a complex programme, the Health Precinct is intended to benefit individuals and their communities through a number of strategies. One strategy is to promote social participation, which enhances an individual’s autonomy and thus maximises their opportunity to make informed choices, which in turn enables co-production and independence [14]. It uses Independent Care Funds from the Conwy County Borough Council and is part of a wider public service that focuses on tackling inequalities and improving outcomes for the poorest. The concept of an integrated hub such as the Health Precinct is not new; two prominent examples are the Bromley by Bow Centre [15], an integrated medical and community hub in London which celebrated its 35 year anniversary in 2019; and the Nuka System of Care in Alaska [16] which promotes community ownership of health services.

If social participation and community assets such as the Health Precinct are to be improved, there is a need to evaluate such initiatives to understand what works and for whom, and to evidence the value generated by these initiatives.

## 2. Materials and Methods

### 2.1. Evaluation Framework–Social Return on Investment Analysis

One of the most widely used forms of economic evaluation is cost-effectiveness analysis, where the incremental costs and effects arising from alternative options, typically an intervention and a control condition, are compared. Effects are expressed in health-related units e.g., lives saved, blood pressure reduction or Quality-adjusted Life Years (QALYs) [17]. The Health Precinct is an established community asset, so it was not deemed ethical to form an artificial control condition by randomizing people with chronic conditions to receive the Health Precinct services immediately or go onto a waiting list. Initiatives such as the Health Precinct are designed to yield both health and social benefits, with outcomes accruing across multiple sectors (health care, social care, local government). As such, a form of cost-benefit analysis, where all inputs and outputs are converted into monetary values, was decided to be more appropriate for the evaluation. This is in line with the National Institute for Health and Care Excellence (NICE) public health guidance, which states that “public health has aspects that are wider than health alone, and these are more readily recognised in a local government environment. This necessitates both making the method of analysis more inclusive, and a corresponding change in perspective” [18]. Cost-consequence analysis and cost-benefit analysis are noted by NICE as methods that can be appropriate for the evaluation of public health interventions. Likewise, the Treasury guidance to appraisal and evaluation in central government notes that social cost-benefit analysis requires “all impacts–social, economic, environmental, financial etc. to be assessed relative to continuing with what would have taken place in the absence of intervention” [19].

Due to the lack of control condition, and the nature of health and social benefits that were expected to accrue across sectors, it was decided to use Social Return on Investment (SROI) analysis to evaluate the Health Precinct. SROI analysis is a form of cost-benefit analysis that considers the social value generated by an initiative, considering a triple bottom line of social, economic and environmental value [20]. SROI analysis has been used in the education and transport sectors historically, and is increasingly being used in health and social care evaluations [21,22]. A societal perspective was undertaken to reflect the multiple stakeholder groups to whom costs and benefits arise to.

Ethical approval for this study was granted by the Wales Research Ethics Committee 4 (ref 17/WA/0122) on 3 May 2017. Reporting follows the Hutchinson et al. [21] SROI quality framework.

### 2.2. Participants–Establishing Scope and Identifying Stakeholders

The initial stage of an SROI analysis involves setting the scope of the evaluation and defining who the relevant stakeholders are. Stakeholders are groups of people or organizational bodies on which the Health Precinct would be expected to materially impact on. The stakeholders were determined to be: Health Precinct participants aged 55 and over, family members of participants, NHS Wales (who co-fund the initiative and could expect to observe a reduction in service use by participants who complete a programme) and Conwy County Borough Council (who co-fund the initiative and take a lead on organisation and delivery). The following inclusion criteria were set for the primary stakeholders, Health Precinct participants:

- Aged 55+
- Referred to the Health Precinct and attended a minimum of one consultation
- Living at home i.e. not a resident in nursing or residential care
- No or mild cognitive impairment, as measured by the 6-CIT test [23].

### 2.3. Theory of Change–Mapping Outcomes

**Figure 1:**
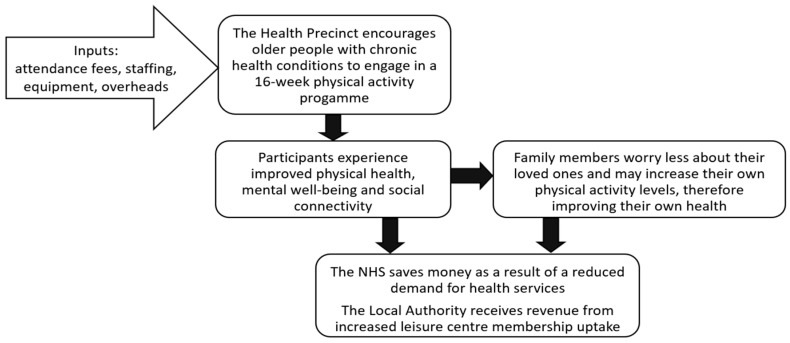
Overview of the theory of change.

As part of a separate realist evaluation work-stream for this study (which aimed to establish what works about the Health Precinct, for whom, and in what circumstances), two focus groups were held with staff involved with the organisation and delivery of the Health Precinct (n = 10) to develop preliminary theories about how and why the Health Precinct brings about change for the various stakeholders. Individual interviews were then held with 16 people aged 55+, with chronic conditions, who had experience of attending the Health Precinct to test and refine the theories. Theories arising from the focus groups and interviews were subsequently used to develop a theory of change for the SROI analysis (Figure 1). The theory of change took a multi-stakeholder perspective and described the process of how inputs lead to material changes and various outcomes for each stakeholder group, thus informing the selection of outcome measures to include in questionnaire packs.

### 2.4. Data Collection–Evidencing Outcomes

All people referred to the Health Precinct between October 2017 and May 2019, and who met the inclusion criteria described in Section 2.3, were invited to take part in the study. Consenting participants received a participant information sheet and a baseline questionnaire at their initial consultation meeting. Informed consent was obtained from all participants who took part in the study. Follow-up questionnaires were sent by post 16 weeks later—the typical length of a participant’s individual plan. Data were entered in Excel version 1902 (Microsoft, Redmond, WA, USA) and analysed in SPSS version 25 (I.B.M., Armonk, NY, USA). Participant questionnaires included:

- Demographic information
- Health conditions and reason for referral
- Health and social care resource use in the past 16 weeks
- Rosenberg Self-Esteem scale [24]
- Campaign to End Loneliness Scale [25]
- EQ-5D-5L [26]

Family members of participants completed postal questionnaires at the 16-week point. These questionnaires contained rating scale questions on family members’ own health and well-being, and how they attributed their loved one’s attendance at the Health Precinct to have impacted on them.

Table 1 describes the threshold criteria that was set for a material change to have occurred for each outcome. Some of the threshold criteria were objective (e.g., increased physical activity was deemed to have occurred if at the end of a 16-week programme participants self-reported meeting the NHS guidance of 150 min of moderate intensity activity per week), while some were subjective (e.g., higher confidence was deemed to have occurred if participants reported an improved score on the Rosenberg Self-Esteem scale between baseline and follow-up).

### 2.5. Valuing Inputs and Outcomes

The costs of inputs required to run the Health Precinct for one year were provided by a programme administrator in 2018 prices. These costs included: attendance fees paid by participants, staffing costs (covering Health Precinct staff and a proportion of general staffing for the leisure centre e.g., receptionists), leisure centre overheads, and exercise equipment annuitized over 12 years at a discount rate of 3.5%.

A range of sources were used to assign a monetary value to outcomes. The main source was the HACT Social Value Calculator version 4 [27], which uses well-being valuation on national surveys to isolate the effect of a factor (e.g., confidence) on an individual’s well-being and identify what the equivalent amount of money required to increase well-being by the same amount would be [28]. National unit costs were used to cost GP appointments [29]. Table 1 lists the monetary valuation source for each outcome.

### 2.6. Establishing Impact (Attribution, Deadweight, Displacement, Drop-Off)

To minimise the risk of overclaiming the benefits it was necessary to account for deadweight, displacement, attribution and attrition. Deadweight is the proportion of observed change that stakeholders would experience over the study period, regardless of taking part in the Health Precinct. Displacement refers to the potential outcomes that are being displaced by the Health Precinct e.g., participants giving up other activities in order to attend the Health Precinct. Attribution refers to the proportion of observed changes that we can confidently say are due to taking part in the Health Precinct as opposed to change resulting from stakeholders taking part in other activities. Drop-off refers to the proportion of outcomes that will be lost after one year. Rating-scale questions were included in the follow-up questionnaires for Health Precinct participants and their families to elicit the deadweight, displacement, attribution and attrition percentages to offset against each outcome. For example, family members were asked a rating scale question to establish what proportion of the change in their own health status they believed was due to their loved one taking part in the Health Precinct. If they answered ‘None’, an attribution percentage of 100% of the outcome being caused by other activities was applied. If they answered ‘a little’, a percentage of 75% was applied; for ‘some’, a percentage of 50% was applied, and for ‘a substantial amount’ a percentage of 25% of the outcome arising from elsewhere was applied. Responses to these questions were aggregated and the mean response used as the proportion to include in the analysis for the stakeholder group.

## 3. Results

Baseline data were collected for 159 Health Precinct participants, and follow-up data were available for 66 participants (see Table 2). More women than men attended an initial consultation for the Health Precinct (97 compared to 62). Programme completers were slightly older than non-completers (73.8 compared to 71.4, a non-significant difference). The most frequent pathway that people were referred to was the National Exercise Referral Scheme (NERS), a programme which has previously been shown to increase physical activity for people with coronary heart disease, and reduce anxiety and depression in people referred for mental health reasons [30].

### 3.1. Inputs

The largest cost category for inputs was staffing. In addition to programme-specific staff, it was assumed that 5% of leisure centre receptionists’ time was spent dealing with Health Precinct participants e.g., taking bookings and processing payments, and 5% of general exercise assistants’ time was spent supporting Health Precinct participants. The total cost of staffing over a year was £45,331.

Overheads such as utilities and facilities, were assumed to be 1.6% of the leisure centre’s total overheads on the basis of Health Precinct sessions operating during 16% of the centre’s opening hours and taking 10% of leisure centre space during this time. The cost of overheads assigned to the Health Precinct was £5998. Equipment was bought especially for the Health Precinct and requires replacing every twelve years. Assuming a depreciation rate of 3.5% per year, the average annual cost of equipment was £1552.

Participants attended an average of 19 sessions over their 16-week programme and paid £2 per session to attend, leading to a financial input of £2508. On completion of their programme, participants were offered the opportunity to purchase annual leisure centre membership discounted by 40%. To avoid double counting, this was not included as an input as it was included as a monetary benefit for Conwy County Borough Council (the local government stakeholder group) on the outcome side of the cost-benefit ratio instead.

The total cost of Health Precinct inputs for one year was calculated to be £55,389.

### 3.2. Outputs, Outcomes and Social Value

To be able to quantify change, data were only included in the SROI analysis for participants who completed both a baseline and follow-up questionnaire (n = 66). Family member data were available for 38 people. Using percentages elicited from stakeholder questionnaires, a deadweight proportion of 25% and a drop-off proportion of 50% was applied to all outcomes. The attribution proportion varied by stakeholder group, with participant outcomes attributed to other activities by 50%, family member outcomes attributed to other activities by 75% and the NHS and local government outcomes attributed to other activities by 25%. No displacement was reported for any stakeholder group. Outcomes were assumed to last for one year in the base case scenario. No unintended outcomes were observed.

Table 3 shows the number of people experiencing material changes for each outcome, and what the resulting social value generated was once deadweight, attribution and drop-off percentages were applied. In total, £281,010 of social value was generated by the Health Precinct in a one-year period. Whilst there were other outcomes which affected more people, the outcome which generated the most social value was an improvement in participant health (£98,187), followed by an improvement in family member health (£45,317). Both outcomes were assigned a monetary value of £20,141 per person affected, which was the highest financial proxy assigned to any outcome.

### 3.3. Social Return on Investment Ratio

Dividing the social value of benefits experienced by stakeholders (£281,010) by the value of inputs required to deliver the Health Precinct (£55,389) yielded a base case SROI ratio of £5.07 of social value generated for every £1 spent.

### 3.4. Sensitivity Analysis

A series of sensitivity analyses were undertaken to test the robustness of the assumptions underpinning the base case scenario (Table 4). The lowest SROI ratio of 2.60:1 resulted from a scenario where different financial proxies were selected for the outcomes with the two highest values: improvement in health status (changed from base case of £20,141 per person to £10,220 per person), and higher confidence (changed from base case of £13,080 per person to £1314 per person). The highest SROI ratio of 5.16:1 resulted from a scenario where social connection was assumed to have occurred in 50% more participants than it was observed in compared to the base case. The small range across SROI ratios in the sensitivity analysis suggests that the base case scenario of 5.07:1 is robust.

## 4. Discussion

Cost-benefit analysis is a useful, and NICE recommended, evaluation method for public initiatives [18,19]. This study used Social Return on Investment (SROI) analysis to evaluate the social value generated by the Health Precinct, a community hub which encourages participants to manage chronic conditions through social prescribing to physical activity and social participation programmes. Social Return on Investment analysis has noted methodological limitations, including lack of equity weightings, variation in the methods used to value outcomes, and lack of guidance on how to interpret SROI ratios [31]. However, when the outcomes from an intervention go beyond health and accrue to multiple stakeholder groups it has advantages over other evaluative frameworks; for instance in a cost-utility analysis the measure of effect is health-related quality of life, therefore the value of the social aspects of an intervention may not be fully captured. The use of sensitivity analyses in this study to vary the financial proxies used in the base case scenario and to vary the assumptions made about attribution and length of equipment lifespan allows for different scenarios to be considered, going some way towards overcoming the limitation of variation in methods used to value outcomes by different SROI practitioners. To examine the effect of using SROI methodology, future research studies of interventions which are expected to have an impact on physical health, well-being and social participation, have the potential of including multiple evaluation methods (e.g., cost-utility alongside SROI analysis) to explore how the findings from the different methods vary.

The stakeholders included in our analysis were Health Precinct participants, family members of participants, NHS Wales and Conwy County Borough Council. A base case SROI ratio of £5.07 of social value generated for every £1 invested was estimated, with sensitivity analysis yielding a range of £2.60 to £5.16. The findings suggested that social value was generated to participants through an increase in their physical activity levels, improvements in health status, increased confidence and increased social connectivity, which is aligned with the outcomes expected from the theory of change that was developed. The theory of change hypothesized that family members of Health Precinct participants would experience improvements in their own health status due to increasing their own physical activity levels and being less worried about their loved ones’ health and well-being. Whilst the SROI analysis found that social value was indeed generated for family members, the questionnaires revealed that participants only attributed 25% of this change in well-being as being due to their loved ones attending the Health Precinct. The two stakeholder groups who provided the highest level of financial contribution into the running of the Health Precinct, NHS Wales and Conwy County Borough Council, received the lowest amount of social value in return (£4069). However, as the concept of an integrated hub such as the Health Precinct is to improve health and well-being for the wider community it may be appropriate that participants and their families are the main beneficiaries. It was expected from the theory of change that there would be an increase in leisure centre membership uptake following attendance at a 16-week Health Precinct programme; however, at the end of the study less than half of participants indicated that they would be interested in taking up annual membership at a discounted rate. Reasons for this included participants purchasing their own gym equipment to be able to exercise at home, and participants intending to maintain their physical activity levels through activities that did not require leisure centre attendance e.g., walking outdoors, or doing home-based exercises that did not require equipment. These reasons suggest that the aim of the Health Precinct to promote independence and increase participants’ autonomy were successful.

Few rigorous, high quality, SROI analyses have been published of social prescribing and health and social care interventions [21,22]. A report on the social value of the Healthy Living Wessex’s Activate Your Life intervention to promote healthier lifestyles to people with weight-related issues through group and one-to-one mentoring estimated an SROI ratio of £5.42: £1 [32]. Their stakeholders included adult and child participants, employers and health and social care services. Whilst the Health Precinct evaluation did not collect data on employment status, the mean age for participants on enrollment to the scheme was 72.4 (SD 8.3)—thus employers were not included as a stakeholder as we did not expect many participants to be in employment. A study of moving services for people with osteoarthritis from a clinical general practitioner led model to a physiotherapy led model yielded an SROI range of between £2.43 and £4.03 for every £1, with participant outcomes of increased levels of physical activity, improved physical and mental health, reduced pain and a reduction in time spent travelling to GP clinics. The NHS benefitted through reduced GP consultations and secondary referrals [33]. With regards to interventions targeting social isolation, an SROI analysis of peer support for people with dementia estimated an SROI ratio of between £1.17 and £5.18 for every £1 invested [34]. Stakeholders included participants, their carers, and the volunteer facilitators for the peer support groups. Participants experienced a reduction in loneliness and increased mental stimulation, carers experienced reduced stress and burden, and volunteers felt fulfilled and had an increased knowledge of dementia. Although none of the interventions in the aforementioned studies are strictly equivalent to the Health Precinct, there is a connection through initiatives that target physical activity and social participation. The SROI ratio for the Health Precinct (£5.07: £1) is in the same region as the SROI ratios generated by these other schemes.

A strength of this evaluation is the societal perspective that it adopts, and the inclusion of multiple stakeholder groups. The theory of change underpinning the selection of outcome measures was robustly developed following a process of stakeholder focus groups and participant interviews. A limitation is that family member data were only available for just over half (38/66) of participants who completed a 16-week Health Precinct programme; however, as a quarter of participants lived by themselves it is possible that they did not feel it relevant to pass on a questionnaire pack to a more distant family member. The retention rate of research participants between baseline and 16-weeks (42%) is consistent with drop-out rates for the Health Precinct programme (47% of people attending an initial consultation go on to complete a 16-week programme). The methods used in this study are applicable to evaluations of a range of public health initiatives, particularly those targeting outcomes beyond physical health.

## 5. Conclusions

The amount of social value generated by the Health Precinct outweighed the cost of inputs required to deliver their programmes. Value was generated to people who were prescribed to the programme, their families, the NHS, and the local government.

Local governments and Health Boards are increasingly interested in evaluation methods which capture the broader outcomes beyond health benefits arising from initiatives, such as added social value. Taking a societal perspective allows for the costs and benefits to a wide group of stakeholders to be considered, beyond the narrow patient perspective (which focuses on costs and outcomes relevant to the participant) or service provider perspective (which focuses on costs and outcomes relevant to the service provider). This societal approach is useful to organisations, such as local authorities, which have budgets that need to be allocated between various sectors in their jurisdiction.

Methods such as cost-benefit analysis and SROI analysis assign a monetary value to outcomes accruing to multiple stakeholder groups, allowing findings to be reported in a common metric (£s). Using a straight-forward ratio of value generated relative to value of inputs to present the results of a study has the benefit of making the findings easier to interpret and understandable to a wide audience, such as service commissioners and providers seeking evidence on social prescribing, community hubs and physical activity programmes. However, the underlying value of conducting an SROI analysis is that it develops a theory of change which explores how value is generated for various stakeholders, and therefore how an organisation can measures changes which are relevant to the people or groups who are materially affected by their activities.

## Figures and Tables

**Table 1:** Stakeholder outcomes and how material changes were defined and valued.

Stakeholder	Outcome	Material Change Defined by	Monetary Valuation Source	Value
Health Precinct participants	Increased physical activity	Self-reporting 150+ min of exercise per week	HACT Social Value Calculator v4: Frequent moderate exercise	£4179
	Improvement in health status	Improvement of >0.1 on the EQ-5D-5L	HACT Social Value Calculator v4: Good overall health	£20,141
	Higher confidence	Improvement in Rosenberg Self-Esteem score	HACT Social Value Calculator v4: High confidence (adult)	£13,080
	Increased social connection	Improvement in Campaign to End Loneliness Scale score	HACT Social Value Calculator v4: Member of a social group	£1850
Family members	Improvement in health status	Improvement of 1 level or more on a 11 level self-reported health status scale	HACT Social Value Calculator v4: Good overall health	£20,141
NHS Wales	Reduction in GP attendances	Difference between number of GP appointments in the 16 weeks before baseline and the 16 weeks of the programme	Unit cost of a GP appointment	£37
Conwy County Borough Council	Increase in leisure centre membership	Number of people reporting that they would take up annual membership at 16 weeks	Annual cost of discounted membership	£233

**Table 2:** Participant characteristics at baseline.

	Number (%), Unless Described Otherwise		
Characteristic	All Participants (n = 159)	Programme Completers (n = 66)	Programme Non-Completers (n = 93)
Age	Mean age 72.4 (SD 8.3) Min–max (55–94)	Mean age 73.8 (SD 8.9) Min–max (55–94)	Mean age 71.4 (SD 7.8) Min–max (56–90)
Men Women	62 (39.0%) 97 (61.0%)	28 (42.4%) 38 (57.6%)	34 (36.6%) 59 (63.4%)
Self-reported health status:			
Excellent	2 (1.3%)	1 (1.5%)	1 (1.1%)
Good	68 (42.8%)	30 (45.5%)	38 (40.9%)
Fair	65 (40.9%)	26 (39.4%)	39 (41.1%)
Poor	19 (11.9%)	7 (10.6%)	12 (12.9%)
Missing	5 (3.1%)	2 (3.0%)	3 (3.2%)
Living situation:			
Living with others	112 (70.4%)	49 (74.2%)	63 (67.7%)
Living alone	47 (29.6%)	17 (25.8%)	30 (32.3%)
Programme pathway:			
NERS	124 (78.0%)	49 (74.2%)	75 (80.6%)
Lifestyle Management	18 (11.3%)	11 (16.7%)	7 (7.5%)
Well-being	1 (0.6%)	1 (1.5%)	0
Cardiac Rehab	6 (3.8%)	4 (6.1%)	2 (2.2%)
Missing	10 (6.3%)	1 (1.5%)	9 (9.7%)

**Table 3:** Social value generated by each stakeholder group.

Stakeholder	Outcome	Financial Proxy	Number Experiencing the Outcome	Deadweight	Attribution to Other Reasons	Drop-Off	Net Social Value
Health Precinct participants (n = 66)	Frequent physical activity	£4179	32	25%	50%	50%	£50,148
	Improvement in health status	£20,141	13	25%	50%	50%	£98,187
	Higher confidence	£13,080	15	25%	50%	50%	£73,575
	Increased social connection	£1850	14	25%	50%	50%	£9713
Family members (n = 38)	Improvement in health status	£20,141	12	25%	75%	50%	£45,317
NHS Wales	Reduction in the number of GP attendances *	£37	19 *	25%	25%	50%	£400
Conwy County Borough Council	Increase in leisure centre membership	£233	28	25%	25%	50%	£3670
						TOTAL	£281,010

* Refers to the reduction in GP appointments rather than the number of people experiencing the outcome.

**Table 4:** Sensitivity analysis.

Base Case	Revised Scenario	Revised Ratio
Financial proxies from the HACT Social Value Bank: improvement in health status is £20,141 per person; high confidence is £13,080 per person	Financial proxies from the Global Value Exchange: improvement in health status is £10,220 per person (value for relief from health problems that limit daily activities); confidence financial proxy is £1314 per person (self-esteem change)	2.60:1
Participant health status improvement measured by a > 0.1 improvement in EQ-5D-5L score (n = 13)	Participant health status improvement measured by a > 0.2 improvement in EQ-5D-5L score (n = 5)	3.98:1
Participants’ improvement in health attributed to Health Precinct at 50%	Participants’ improvement in health attributed to Health Precinct at 25%	4.19:1
Equipment replaced every 12 years	Equipment replaced every 5 years	4.92:1
Assumption that all people who said they would take up leisure centre membership will do so (n = 28)	Assumption that only half of those who said they would take up leisure centre membership will do so (n = 14)	5.04:1
Increased social connection reported in 14 participants	Increased social connection experienced by +50% more participants (n = 21)	5.16:1
